# Effect of Red Ginseng on Genotoxicity and Health-Related Quality of Life after Adjuvant Chemotherapy in Patients with Epithelial Ovarian Cancer: A Randomized, Double Blind, Placebo-Controlled Trial

**DOI:** 10.3390/nu9070772

**Published:** 2017-07-19

**Authors:** Hee Seung Kim, Mi-Kyung Kim, Maria Lee, Byung-Su Kwon, Dong Hoon Suh, Yong Sang Song

**Affiliations:** 1 Department of Obstetrics and Gynecology, Seoul National University College of Medicine, Seoul 03080, Korea; bboddi0311@gmail.com (H.S.K.); marialeemd@gmail.com (M.L.); 2 Department of Obstetrics and Gynecology, Cheil General Hospital & Women’s Healthcare Center, Dankook University College of Medicine, Seoul 04619, Korea; asterik79@gmail.com; 3 Department of Obstetrics and Gynecology, Pusan National University School of Medicine, Busan 49241, Korea; kbs866@naver.com; 4 Department of Obstetrics and Gynecology, Seoul National University Bundang Hospital, Seongnam 13620, Korea; sdhwcj@naver.com; 5 Cancer Research Institute, Seoul National University College of Medicine, Seoul 110-799, Korea

**Keywords:** red ginseng, toxicity, health-related quality of life, chemotherapy, epithelial ovarian cancer

## Abstract

We evaluated the effect of red ginseng on toxicity, health-related quality of life (HRQL) and survival after adjuvant chemotherapy in patients with epithelial ovarian cancer (EOC). A total of 30 patients with EOC were randomly assigned to placebo (*n* = 15) and red ginseng groups (*n* = 15). All patients took placebo or red ginseng (3000 mg/day) for three months. Then, we compared changes of genotoxicity, HRQL and survival between the two groups. As a result, red ginseng reduced micronuclei yield in comparison with placebo despite no difference of binucleated cells index. Although red ginseng increased serum levels of alanine aminotransferase and aspartate aminotransferase significantly, they were within the normal value. Moreover, there were no differences in adverse events between placebo and red ginseng groups. In terms of HRQL, red ginseng was associated with improved emotional functioning and decreased symptoms of fatigue, nausea and vomiting, and dyspnea, reduced anxiety and interference affecting life and improved daytime somnolence. However, there was no effect of red ginseng on prognosis of EOC. Conclusively, red ginseng may be safe and effective to reduce genotoxicity and improve HRQL despite no benefit of survival in patients with EOC who received chemotherapy.

## 1. Introduction

Epithelial ovarian cancer (EOC) is the most lethal disease among malignancies developed in the female genital tract because of no effective screening method for early detection. Thus, about 80% of patients are diagnosed at advanced-stage disease, which leads to poor prognosis [1]. So, many relevant basic research and clinical trials are focused on prolongation of survival by new drugs overcoming chemo-resistance in EOC.

In addition to oncologic outcomes, psychological stress as well as reduced physical activity are also very important during the treatment of EOC because they lead to poor health-related quality of life (HRQL) including cancer-related fatigue, and reduce physical and social function, which consequently has an influence on the prognosis of patients with EOC [2,3,4]. Moreover, poor HRQL is known not to be reduced by rest or sleep but to be worsened by chemotherapy in cancer patients [3], and 70% of EOC patients treated with chemotherapy are known to experience poor HRQL commonly [5].

Underlying mechanisms of poor HRQL by chemotherapy are as follows. First, most of chemotherapeutic agents not specifically targeted against neoplastic cells but also affect normal cells, which results in various adverse reactions [6]. Moreover, a developed state of oxidative stress, directly or indirectly caused by numerous drugs, induces various types of genotoxicity such as DNA adducts, gene mutations and chromosomal aberrations [7].

For overcoming poor HRQL induced by chemotherapy, the interest in complementary and alternative medicine such herb has increased up to now [8]. In particular, ginseng has been shown to have beneficial effects on the central nervous, endocrine, cardiovascular and immune systems as a potential biological response modifier [9,10,11,12,13]. Some studies have shown that ginseng may have the potential activity of chemoprevention or treatment for stomach, liver, pancreas and colon cancers by inhibiting the inflammation to cancer sequence [14]. Moreover, ginseng showed the advantages in terms of increasing the sensitivity of chemotherapy or radiotherapy, which reduced adverse effects and improved quality of life and prognosis [15]. However, there is still a lack of clinical evidence showing the effect of ginseng in cancer patients.

Thus, we performed a randomized, double blind, placebo-controlled trial for evaluating the effect of red ginseng, the product of ginseng processed by steaming, on genotoxicity and relevant HRQL in patients with EOC who received chemotherapy.

## 2. Materials and Methods

### 2.1. Patients

We conducted the current study at Seoul National University Hospital from April 2012 to November 2014, and the relevant protocol was approved by the Institutional Review Board (No. 1208-131-423). We enrolled patients who met the following criteria: those with EOC over 20 years old; those who received six cycles of adjuvant taxane- and platinum-based chemotherapy after cytoreductive surgery; those who showed complete or partial response after the treatment; those who completed the treatment within eight weeks prior to initiating the current study. However, we excluded patients with a history of smoking, chronic disease such as diabetes mellitus, recurrent disease requiring more intensive treatment, previous radiotherapy, a history of hypersensitivity to ginseng, ginseng consumption during treatment.

### 2.2. Study Design

The current study was a randomized, double blind, placebo-controlled trial for 12 weeks. For the current design, we prepared two types of soft capsules including 500 mg of red ginseng or 500 mg of corn starch as placebo provided the Korean Ginseng Cooperation (Dajeon, Korea). The Korean red ginseng capsule was manufactured from roots of a 6-year-old red ginseng, Panax ginseng Meyer, harvested in our country. It was made by steaming fresh ginseng at 90–100 °C for 2 to 3 h and then drying at 50–80 °C. Finally, the capsule including powder (500 mg/capsule) was prepared from grinded red ginseng. The Korean red ginseng contained major ginsenoside-Rb1 (5.61 mg/g), -Rb2 (2.03 mg/g), -Rc (2.20 mg/g), -Rd (0.39 mg/g), -Re (1.88 mg/g), -Rf (0.89 mg/g), -Rg1 (3.06 mg/g), -Rg2(s) (0.15 mg/g), -Rg3(s) (0.17 mg/g), -Rg3(r) (0.08 mg/g), -Rh1 (0.30 mg/g), and other minor ginsenosides.

After we obtained written informed consents, all patients were randomly assigned to red ginseng or placebo groups, and took two capsules three times a day (a total of 3000 mg/day) for three months. During the current study, all patients visited twice at our center, and the five-item subscales related with HRQL and blood sampling for evaluating toxicity were conducted (week 0 and week 12) like previous studies [16,17]. Moreover, we evaluated compliance with consumption of red ginseng or placebo at the end of the study by counting the remaining capsules, and patients were excluded at the final analysis if their compliance rate was less than 75%.

### 2.3. Toxicity

All patients were asked to list adverse events after consumption of red ginseng or placebo according to the National Cancer Institute’s Common Terminology Criteria for Adverse Events version 4 (NCI CTCAE v4). Moreover, we compared hematological and biochemical variables between 0 and 12 weeks in both red ginseng and placebo groups. Specifically, white blood cells (WBC), hemoglobin, platelet, neutrophil and lymphocyte were counted, and serum levels of alanine aminotransferase (ALT), aspartate aminotransferase (AST), bilirubin, alkaline phosphatase (ALP), blood urea nitrogen (BUN) and creatinine were evaluated at both week 0 and week 12.

For evaluating genotoxicity, we performed the cytokinesis block micronucleus (CBMN) assay in both groups. The peripheral venous blood samples were collected into heparinized tubes at two different times (week 0 and week 12), and we isolated lymphocytes by the density gradient centrifugation technique according to the method by Fenech [18]. Then, we estimated binucleated cells (BN) index and micronuclei (MN) yield for assess drug-induced genotoxicity. BN index was defined as total number of BN/total number of nucleated cells ×1000, and MN yield was determined as total number of BN including MN/total number of BN ×1000. The two values were counted by using the IMSTAR system for automatic image analysis of MN [19].

### 2.4. Health-Related Quality of Life

We evaluated the effect of red ginseng or placebo on HRQL using the five-item subscales including the European Organization for Research and Treatment of Cancer QLQ-C30 (EORTC QLQ-C30), Brief Fatigue Inventory (BFI), Brief Pain Inventory (BPI), Hospital Anxiety and Depression Scale (HADS) and Sleep Scale from the Medical Outcome Study (MOS-SS) [20,21,22,23,24].

The EORTC QLQ-C30 incorporates five functional scales (physical, role, cognitive, emotional, and social), global quality of life and nine symptom scales (fatigue, nausea and vomiting, pain, dyspnea, sleep disturbance, appetite loss, constipation, diarrhea and financial problem). All items are scored from 1 (not at all) to 4 (very much), with the exception of two items of global quality of life scored by using modified 7-point linear analog scales [20].

The BFI includes nine items which represents fatigue and its interference measured on numeric scales from 0 to 10. The first three items evaluate the severity of fatigue at “right now”, at “usual level”, and at the worst level during the previous 24 h. The next six items represent how much fatigue interferes with some aspects of life (general activity, mood, walking, ability, normal work, relation with other people, and enjoyment of life) during the previous 24 h. The interference is measured as the sum of the six items, which range from 0 to 60 [21].

The BPI is a method for evaluating pain which measures the intensity of pain and the degree to which pain interferes with a patient’s life. It asks patients to rate their pain at its current intensity, at its worst, least, and average over the previous week. The BPI uses an 11-point numeric rating scales (0, no pain; 10, pain as bad as you can imagine to investigate the intensity of pain. Moreover, it also asks patients to rate how their pain interferes with general activity, mood, walking, work, sleep, social relation, and enjoyment of life by using a similar numeric rating scale (0, no interference; 10, interferes completely) [22].

The HADS is a tool to assess the symptoms of anxiety and depression. The 14-item questionnaire includes seven questions related to depression and seven relating to anxiety. Each question is scored based on a four-point scale ranging from 0 (not all all) to 3 (very often). Higher mean values of each seven questions mean greater likelihoods of anxiety and depression [23]. The MOS-SS yields a sleep problems index and six scale scores such as sleep disturbance, daytime somnolence, sleep adequacy, snoring, awaken short of breath or with headache and quantity of sleep. Quantity of sleep is scored as the mean hours slept per night. The other scales and sleep problems index are scored on a 0 to 100 possible range [24].

### 2.5. Survival

We evaluated the effect of red ginseng on survival in patients with EOC. Progression-free survival (PFS) was defined as the time from the date of diagnosis to the date of recurrence. Overall survival (OS) was considered as the time from the date of diagnosis to the date of cancer-related death or end of the study.

### 2.6. Statistical Analyses

The sample sized was calculated using Power Analysis and Sample Size (PASS) 15 software [25]. The level of significance was set to 5% (two-sided), and the power to 80%. We estimated that a minimum sample size of 34 patients was required, considering mean and standard deviation of MN frequency before and after consumption in previous studies [26,27], and dropout rate of 10%. The primary endpoint was to compare BN index and MN yield between week 0 and week 12, and the secondary endpoints were to evaluate adverse events after consumption of red ginseng and its impact on HRQL and survival. For these statistical analyses, we used Wilcoxon signed rank, Chi square or Fisher exact, and Kaplan-Meier with the log rank tests in SPSS software version 21.0 (SPSS Inc., Chicago, IL, USA), and a *p* < 0.05 was considered to be statistically significant.

## 3. Results

### 3.1. Patients’ Characteristics

All 34 patients received the screening test for the current study. However, four patients discontinued the study due to personal reasons (*n* = 3) and suspicious recurrence (*n* = 1). Thus, a total of 30 patients were enrolled, and randomly assigned to red ginseng (*n* = 15) and placebo groups (*n* = 15) (Supplementary Figure S1). Table 1 shows clinic-pathologic characteristics of the two groups, and there were no differences in age, body mass index, compliance, disease status and histology. Moreover, all patients showed the compliance of more than 90%. Histologically, serous adenocarcinoma was the most common type (*n* = 19), and endometrioid (*n* = 7) and clear cell carcinoma (*n* = 4) were classified as non-serous type.

### 3.2. Toxicity

**Figure 1:**
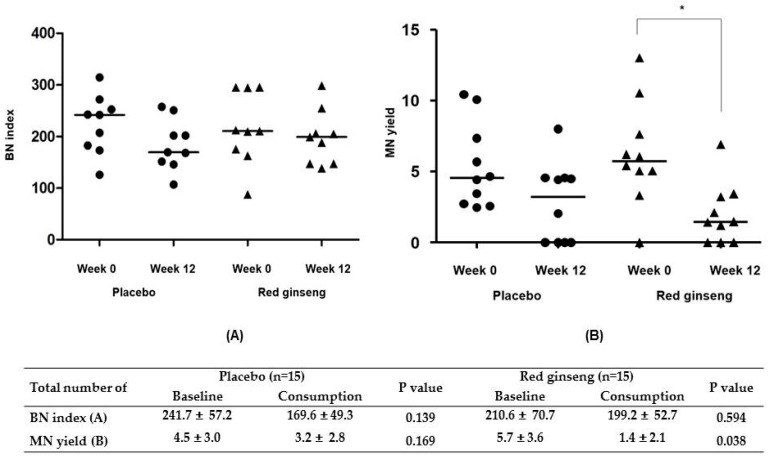
Comparison of (**A**) binucleated cells (BN) index and (**B**) micronuclei (MN) yield between placebo and red ginseng groups.

For evaluating the effect of red ginseng on genotoxicity, we evaluated BN index and MN yield. As a result, there was no difference of BN index and MN yield between week 0 and week 12 in the placebo group (*p* > 0.05). Although there was no difference in BN index between week 0 and week 12, the median value of MN yield was lower at week 12 than week 0 (median, 1.4 and 5.7; *p* = 0.038) in red ginseng group (Figure 1).

In terms of laboratory markers, lymphocyte and platelet counts, bilirubin, ALP, BUN and creatinine levels were not different between week 0 and week 12 in both placebo and red ginseng groups. On the other hand, WBC and neutrophil counts, hemoglobin levels were higher at week 12 than week 0 in both groups. However, AST and ALT levels increased significantly at week 12 when compared with week 0 in red ginseng group despite no difference of AST and ALT levels between week 0 and 12 in placebo groups (Table 2). In terms of adverse events, we observed grade 1 nausea, insomnia, palpitation, headache and urticarial. However, there were no differences in adverse events between red ginseng and placebo groups (Table 3).

### 3.3. Health-Related Quality of Life

For evaluating the effect of red ginseng or placebo on HRQL, we analyzed the results of the five-item subscales. Supplementary Tables S1–S5 depict the comparison of HRQL between placebo and red ginseng groups by using the EORTC QLQ-C30, BFI, BPI, HADS and MOS-SS. When we selected outcomes which were not affected by placebo but were affected by red ginseng, emotional score (functional scale) and fatigue, nausea and vomiting, and dyspnea scores (symptom scale) in the EROTC QLQ-C30, worst fatigue score (severity) and interference score in the BFI, enjoyment of life score (pain interference) in the BPI and anxiety score in the HADS were lower at week 12 than week 0, whereas daytime somnolence score in the MOS-SS was higher at week 12 than week 0 in red ginseng group in spite of no difference between week 0 and week 12 in placebo group (Table 4).

### 3.4. Survival

**Figure 2:**
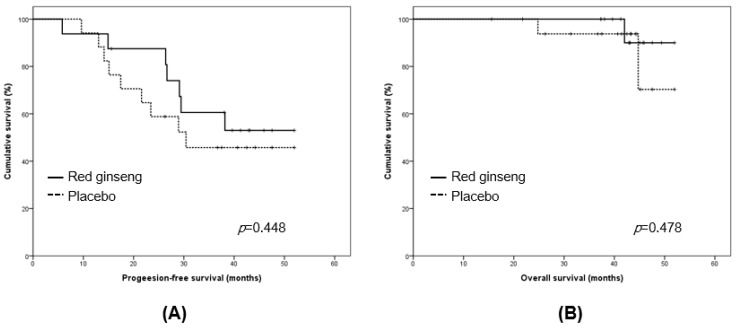
Comparison of (**A**) progression-free survival and (**B**) overall survival between placebo and red ginseng groups.

When we compared PFS and OS between placebo and red ginseng groups, the mean values of PFS were 34.4 and 39.2 months in placebo and red ginseng groups, showing no difference (*p* = 0.448). OS was also not different in placebo and red ginseng groups (mean values, 48.6 vs. 50.9 months; *p* = 0.478; Figure 2).

## 4. Discussion

The ginseng family including Asian ginseng, American ginseng and notoginseng is a commonly used herbal medicine [28]. In particular, Asian ginseng is commercially available as white and red products, and white ginseng is prepared by air-drying after harvest while red ginseng is made by steaming or heating process using fresh ginseng [29]. In red ginseng, various components such as ginsenosides, polysaccharides, peptides, polyacetylenic alcohols and fatty acids are included [30]. Among them, ginsenosides can show most of the pharmacological actions of red ginseng [31]. The activity is known to be related with the reduced genotoxic effects against chemotherapy or radiotherapy [32,33], and the improvement of quality of life in cancer patients [34].

When we think that most of patients with EOC are diagnosed at advanced-stage disease and the recent strategy for treating EOC is focused on cytoreductive surgery with taxane- and platinum-based chemotherapy, poor HRQL may be related with not only the deleterious condition that the disease can exert itself but also the physical and emotional complications induced by chemotherapy in the long term [35]. Thus, we hypothesized that red ginseng could improve quality of life by reducing genotoxicity in patients with EOC who received chemotherapy because previous trials suggested that red ginseng may improve fatigue and cognition, and lead to better quality of life for psychosocial well-being [36].

In the current study, we found that there was no difference of the reduction of BN index between red ginseng and placebo. BN are cells that contain two nuclei, which is commonly found in cancer cells and may arise from a variety of causes. A large portion of BN arising from normal cells remains in interphase and never enter mitosis again, whereas cells that contain mutations before they become binucleate are more likely to proceed through mitosis [37]. Thus, the current study suggests that red ginseng may not decrease BN after chemotherapy when compared with placebo.

On the other hand, red ginseng decreased MN yield significantly when compared with placebo in the current study. In general, MN in mononucleated cells reflect on genetic damage accumulated in vivo during the life span, whereas MN in BN means additional mutations expressed during first in vitro mitosis to the accumulated genetic damage in vivo during the life span [19]. Thus, the current study demonstrates that red ginseng may be effective to reduce additional mutations by decreasing the number of BN including MN when compared with placebo. This fact can be supported by previous studies where MN frequency was higher in cancer patients than in healthy population [26], and low frequency of MN was related with improved survival in cancer patients [38]. Moreover, red ginseng did not increase adverse events in comparison with placebo, and AST and ALT levels were within the normal limit in spite of a significant increase after consumption of red ginseng. Thus, we thought that the safety of red ginseng in the current study may be similar to previous reports [39,40].

Moreover, red ginseng was related with improved HRQL in comparison with placebo in the current study. Specifically, red ginseng was associated with improved emotional functioning and decreased symptoms such as fatigue, nausea and vomiting, and dyspnea in the EORTC QLQ-C30, reduced severity of worst fatigue and interference in the BFI, decreased interference affecting enjoyment of life in the BPI, reduced anxiety in the HADS and improved daytime somnolence in the MOS-SS. These results suggest that red ginseng may improve quality of life after chemotherapy in patients with EOC, which can be supported by previous studies [41,42,43]. Although there is a lack of evidence about underlying mechanisms which link genotoxicity and HRQL in EOC patients, oxidative stress and chronic inflammation is known to be related with public health [44]. Thus, we can hypothesize that oxidative stress or inflammation induced by chemotherapy may reduce HRQL, and red ginseng can improve it by reducing oxidative stress or inflammation represented in preclinical studies [32,45]. Thus, reduced genotoxicity and improved HRQL in the current study support this hypothesis. However, we did not conclude the effect of red ginseng on survival in spite of no difference between placebo and red ginseng groups because the duration of follow up was too short. In some studies, red ginseng has been reported to prolong surgical treatment in gastric and lung cancers [46,47]. When we consider a lack of relevant studies, different tumor biology in solid tumors and various types of treatment, the effect of red ginseng on survival should be further investigated in patients with EOC.

## 5. Conclusions

The current study shows that red ginseng may be effective to reduce genotoxicity and improve HRQL in patients with EOC who received chemotherapy after surgery. Moreover, red ginseng can be taken safely, but it has no effect on improved survival of EOC. However, the current study has a limitations of the small sample size, which can be estimated from a few studies where genotoxicity was investigated. Thus, the results from the current study should be proven in large-scale trials in the future.

## Figures and Tables

**Table 1:** Clinico-pathologic characteristics of patients with epithelial ovarian cancer who received placebo or red ginseng.

Characteristics	Placebo (*n* = 15, %)	Red Ginseng (*n* = 15, %)	*p* Value
Age (year) *	52.9 ± 10.1	55.9 ± 12.1	0.276
Height (year) *	154.8 ± 4.1	155.4 ± 7.8	0.986
Weight (kg) *	56.2 ± 8.7	58.3 ± 8.7	0.363
BMI (kg/m^2^) *	23.5 ± 4.0	24.1 ± 2.8	0.309
Compliance (%) *	93.9 ± 6.7	94.9 ± 6.8	0.599
FIGO stage			1.000
I–II	5 (33.3)	4 (26.7)	
III–IV	10 (66.7)	11 (72.3)	
Histology			0.272
Serous	9 (60)	10 (66.7)	
Non-serous	6 (40)	5 (33.3)	

Abbreviation: BMI, body mass index; CR, complete response; FIGO, International Federation of Gynecology and Obstetrics; PR, partial response; * all values were shown by mean with standard deviation.

**Table 2:** Hematological and biochemical changes after consumption of red ginseng or placebo.

Laboratory Markers	Placebo (*n* = 15)		*p* Value	Red Ginseng (*n* = 15)		*p* Value
	Week 0	Week 12		Week 0	Week 12	
WBC (cells/μL)	4285.7 ± 1944.7	5670.7 ± 2136.3	0.008	4068.8 ± 1304.4	5051.9 ± 1205.4	0.008
Neutrophil (cells/μL)	1989.1 ± 1560.3	2920.0 ± 1629.3	0.030	2057.6 ± 933.1	2804.1 ± 954.9	0.030
Lymphocyte (cells/μL)	1660.5 ± 663.5	2116.0 ± 769.0	0.778	1544.9 ± 538.4	1679.2 ± 597.1	0.163
Hemoglobin	10.2 ± 1.1	12.1 ± 0.8	0.001	10.4 ± 1.0	12.4 ± 0.9	<0.001
Platelet	181.6 ± 86.1	214.8 ± 57.5	0.116	214.9 ± 113.9	209.8 ± 54.9	0.605
AST	28.6 ± 11.5	28.4 ± 14.6	0.834	20.1 ± 7.5	27.4 ± 21.5	0.010
ALT	32.5 ± 20.9	29.4 ± 23.3	0.683	20.2 ± 17.8	29.7 ± 36.8	0.009
Bilirubin	0.5 ± 0.1	0.5 ± 0.1	0.237	0.5 ± 0.2	0.6 ± 0.2	0.070
ALP	67.6 ± 22.5	68.4 ± 27.9	0.925	71.3 ± 15.1	72.8 ± 13.4	0.679
BUN	14.4 ± 5.2	14.9 ± 4.9	0.717	12.8 ± 2.7	12.9 ± 4.1	1.000
Creatinine	0.7 ± 0.1	0.7 ± 0.1	0.363	0.7 ± 0.1	0.7 ± 0.1	0.111

Abbreviation: ALP, alkaline phosphatase; ALT, alanine transaminase; AST, aspartate aminotransferase; BUN, blood urea nitrogen; WBC, white blood cell; all values were shown by mean with standard deviation.

**Table 3:** Adverse events after consumption of red ginseng or placebo.

Adverse Events	Grade	Placebo (*n* = 15, %)	Red Ginseng (*n* = 15, %)	*p* Value
Nausea	1	2 (13.3)	1 (6.7)	1.000
Insomnia	1	1 (6.7)	1 (6.7)	1.000
Palpitation	1	0 (0)	1 (6.7)	1.000
Headache	1	2 (13.3)	1 (6.7)	1.000
Urticaria	1	1 (6.7)	1 (6.7)	1.000
Total No. of patients		3 (20)	2 (13.3)	1.000

**Table 4:** Evaluation of the effect of red ginseng on health-related quality of life.

Outcomes	Placebo (*n* = 15)		*p* Value	Red Ginseng (*n* = 15)		*p* Value
	Week 0	Week 12		Week 0	Week 12	
European Organization for Research and Treatment of Cancer Quality of Life (EORTC QLQ)-C30						
Functional scale						
Emotional	37.5 ± 10.9	38.7 ± 13.9	0.702	44.9 ± 15.3	37.5 ± 12.7	0.027
Symptom scale						
Fatigue	53.7 ± 11.8	45.8 ± 13.9	0.131	58.8 ± 19.2	46.1 ± 16.2	0.012
Nausea and vomiting	30.5 ± 6.4	27.3 ± 5.0	0.157	37.5 ± 15.3	27.2 ± 6.6	0.004
Dyspnea	40.6 ± 22.1	31.3 ± 14.4	0.161	47.1 ± 17.4	35.3 ± 17.8	0.021
Brief Fatigue Inventory (BFI)						
Severity						
Worst fatigue	4.25 ± 2.27	3.62 ± 2.71	0.658	5.59 ± 3.20	4.00 ± 3.32	0.026
Interference	19.25 ± 10.33	11.38 ± 11.84	0.084	23.24 ± 17.79	14.29 ± 17.59	0.014
Brief Pain Inventory (BPI)						
Pain interference						
Enjoyment of life	3.0 ± 3.6	1.8 ± 3.0	0.138	1.8 ±. 2.3	0.4 ± 0.7	0.035
Hospital Anxiety and Depression Scale (HADS)						
Anxiety	7.8 ± 2.4	8.4 ± 1.5	0.119	9.1 ± 2.6	8.0 ± 2.6	0.015
Sleep Scale from the Medical Outcome Study (MOS-SS)						
Daytime somnolence	74.5 ± 14.8	77.5 ± 16.8	0.342	74.7 ± 17.1	83.0 ± 8.9	0.043

All values were shown by mean with standard deviation.

## Supplementary Materials

The following are available online at www.mdpi.com/2072-6643/9/7/772/s1. Figure S1: Flow chart of the study design and subject participation. Table S1: Evaluation of health-related quality of life by using the European Organization for Research and Treatment of Cancer Quality of Life (EORTC QLQ)-C30. Table S2: Evaluation of health-related quality of life by using the Brief Fatigue Inventory (BFI) and BFI scoring system. Table S3: Evaluation of health-related quality of life by using the Brief Pain Inventory (BPI) and BPI scoring system. Table S4: Evaluation of health-related quality of life by using the Hospital Anxiety and Depression Scale (HADS) and HADS scoring system. Table S5: Evaluation of health-related quality of life by using the Sleep Scale from the Medical Outcome Study (MOS-SS).
