# Supplementary material for: Effect of Red Ginseng on Genotoxicity and Health-Related Quality of Life after Adjuvant Chemotherapy in Patients with Epithelial Ovarian Cancer: A Randomized, Double Blind, Placebo-Controlled Trial
Source: Nutrients. 2017 Jul 19;9(7):772. doi: 10.3390/nu9070772 (PMC5537886; doi:10.3390/nu9070772)
Supplement: Supplementary file 1 [file nutrients-09-00772-s001.zip › nutrients-193221-supplementary.pdf]

Supplementary Table S1. Evaluation of health-related quality of life by using the European Organization for Research and Treatment of Cancer Quality of Life (EORTC QLQ)-C30

Outcomes	Placebo (n=15)		*p* value	Red ginseng (n=15)		*p* value
	Week 0	Week 12		Week 0	Week 12	
Functional scale						
Physical	49.4 ± 12.4	40.9 ± 10.4	0.022	54.1 ± 10.6	47.1 ± 15.7	0.018
Role	48.4 ± 18.2	38.3 ± 14.0	0.165	57.4 ± 19.8	48.5 ± 21.1	0.120
Emotional	37.5 ± 10.9	38.7 ± 13.9	0.702	44.9 ± 15.3	37.5 ± 12.7	0.027
Cognitive	40.6 ± 12.5	44.5 ± 12.9	0.218	41.2 ± 12.3	38.2 ± 12.1	0.206
Social	55.5 ± 21.9	42.2 ± 18.2	0.019	61.0 ± 20.7	45.6 ± 23.8	0.012
Global quality of life	63.8 ± 15.5	70.5 ± 19.1	0.178	66.0 ± 15.5	76.1 ± 18.7	0.081
Symptom scale						
Fatigue	53.7 ± 11.8	45.8 ± 13.9	0.131	58.8 ± 19.2	46.1 ± 16.2	0.012
Nausea and vomiting	30.5 ± 6.4	27.3 ± 5.0	0.157	37.5 ± 15.3	27.2 ± 6.6	0.004
Pain	43.0 ± 14.4	36.7 ± 11.6	0.204	50.0 ± 19.8	43.4 ± 17.2	0.179
Dyspnea	40.6 ± 22.1	31.3 ± 14.4	0.161	47.1 ± 17.4	35.3 ± 17.8	0.021
Sleep disturbance	43.8 ± 19.4	40.6 ± 23.9	0.527	41.2 ± 21.5	41.2 ± 17.5	1.000
Appetite loss	37.5 ± 12.9	29.7 ± 10.1	0.025	48.5 ± 20.7	33.8 ± 175	0.031
Constipation	42.2 ± 19.8	40.6 ± 22.1	0.739	33.8 ± 17.5	38.2 ± 23.6	0.180
Diarrhea	29.7 ± 10.1	29.7 ± 10.1	1.000	29.4 ± 13.2	27.9 ± 8.3	0.564
Financial problem	45.3 ± 22.7	42.2 ± 19.8	0.564	50.0 ± 17.7	42.7 ± 19.3	0.132

All values were shown by mean with standard deviation.

Supplementary Table S2. Evaluation of health-related quality of life by using the Brief Fatigue Inventory (BFI) and BFI scoring system

Outcomes	Placebo (n=15)		*p* value	Red ginseng (n=15)		*p* value
	Week 0	Week 12		Week 0	Week 12	
Severity						
Fatigue right now	3.19 ± 1.80	2.44 ± 1.67	0.137	3.71 ± 2.44	2.82 ± 2.48	0.128
Usual fatigue	3.63 ± 1.71	2.31 ± 1.58	0.022	4.24 ± 2.80	2.88 ± 2.47	0.038
Worst fatigue	4.25 ± 2.27	3.62 ± 2.71	0.658	5.59 ± 3.20	4.00 ± 3.32	0.026
Interference	19.25 ± 10.33	11.38 ± 11.84	0.084	23.24 ± 17.79	14.29 ± 17.59	0.014

All values were shown by mean with standard deviation.

BFI scoring system

I. Severity

1. Please rate your fatigue (weariness, tiredness) by circling the one number that best describes your fatigue right now

0 1 2 3 4 5 6 7 8 9 10

No fatigue As bad as you can imagine

2. Please rate your fatigue (weariness, tiredness) by circling the one number that best describes your usual level of fatigue during past 24 hours

0 1 2 3 4 5 6 7 8 9 10

No fatigue As bad as you can imagine

3. Please rate your fatigue (weariness, tiredness) by circling the one number that best describes your worst level of fatigue during past 24 hours

0 1 2 3 4 5 6 7 8 9 10

No fatigue As bad as you can imagine

II. Interference

: Circle the one number that describes how, during the past 24 hours, fatigue has interfered with your: and please sum all scores.

1. General activity

0 1 2 3 4 5 6 7 8 9 10

Do not interfere Completely interfere

2. Mood

0	1	2	3	4	5	6	7	8	9	10

Do not interfere Completely interfere

3. Walking ability

0	1	2	3	4	5	6	7	8	9	10

Do not interfere Completely interfere

4. Normal work (includes both work outside the home and daily chores)

0	1	2	3	4	5	6	7	8	9	10

Do not interfere Completely interfere

5. Relations with other people

0	1	2	3	4	5	6	7	8	9	10

Do not interfere Completely interfere

6. Enjoyment of life

0	1	2	3	4	5	6	7	8	9	10

Do not interfere Completely interfere

Supplementary Table S3. Evaluation of health-related quality of life by using the Brief Pain Inventory (BPI) and BPI scoring system

Outcomes	Placebo (n=15)		*p* value	Red ginseng (n=15)		*p* value
	Week 0	Week 12		Week 0	Week 12	
Pain intensity						
Worst	3.9 ± 3.2	3.2 ± 3.2	0.781	1.8 ± 1.7	1.6 ± 1.6	0.577
Least	2.4 ± 2.3	1.4 ± 1.7	0.152	0.8 ±1.0	0.9 ± 1.9	0.944
Average	3.6 ± 2.8	2.2 ± 2.0	0.080	1.7 ± 1.4	1.3 ± 1.5	0.428
Now	2.8 ± 2.5	1.5 ± 1.9	0.082	1.2 ± 1.4	0.9 ± 1.5	0.671
Pain interference						
General activity	2.7 ± 2.5	2.1 ± 2.8	0.440	1.7 ± 1.9	0.8 ± 1.2	0.139
Mood	2.8 ± 2.5	2.2 ± 2.4	0.301	1.5 ± 1.9	0.9 ± 1.7	0.397
Walking	3.2 ± 2.7	2.2 ± 2.8	0.182	1.6 ± 2.1	0.6 ± 0.9	0.051
Work	2.8 ± 3.0	2.6 ± 2.9	0.686	1.8 ± 2.3	0.7 ± 1.1	0.075
Social relation	2.2 ± 2.9	1.6 ± 2.7	0.101	1.2 ± 1.6	0.5 ± 0.9	0.103
Sleep	2.6 ± 2.5	1.4 ± 2.1	0.057	1.9 ± 2.5	0.5 ± 0.9	0.084
Enjoyment of life	3.0 ± 3.6	1.8 ± 3.0	0.138	1.8 ±. 2.3	0.4 ± 0.7	0.035

All values were shown by mean with standard deviation.

BPI scoring system

I. Pain intensity

1. Please rate your pain by circling nsity the one number that best describes your pain at its worst in the last 24 hours

0 1 2 3 4 5 6 7 8 9 10

No pain Pain as bad as you can imagine

2. Please rate your pain by circling nsity the one number that best describes your pain at its least in the last 24 hours

0 1 2 3 4 5 6 7 8 9 10

No pain Pain as bad as you can imagine

3. Please rate your pain by circling nsity the one number that best describes your pain at the average

0	1	2	3	4	5	6	7	8	9	10

No pain Pain as bad as you can imagine

4. Please rate your pain by circling nsity the one number that tells how much pain you have right now

0	1	2	3	4	5	6	7	8	9	10

No pain Pain as bad as you can imagine

II. Pain interference

: Circle the one number that describes how, during the past 24 hours, pain has interfered with your:

1. General activity

0	1	2	3	4	5	6	7	8	9	10

Does not interfere Completely interferes

2. Mood

0	1	2	3	4	5	6	7	8	9	10

Does not interfere Completely interferes

3. Walking ability

0	1	2	3	4	5	6	7	8	9	10

Does not interfere Completely interferes

4. Normal work (includes both work outside the home and housework)

0	1	2	3	4	5	6	7	8	9	10

Does not interfere Completely interferes

5. Relation with other people

0	1	2	3	4	5	6	7	8	9	10

Does not interfere Completely interferes

6. Sleep

0	1	2	3	4	5	6	7	8	9	10

Does not interfere Completely interferes

7. Enjoyment of life

0	1	2	3	4	5	6	7	8	9	10

Does not interfere Completely interferes

Supplementary Table S4. Evaluation of health-related quality of life by using the Hospital Anxiety and Depression Scale (HADS) and HADS scoring system

Outcomes	Placebo (n=15)		*p* value	Red ginseng (n=15)		*p* value
	Week 0	Week 12		Week 0	Week 12	
Anxiety	7.8 ± 2.4	8.4 ± 1.5	0.119	9.1 ± 2.6	8.0 ± 2.6	0.015
Depression	12.9 ± 2.1	12.4 ± 1.9	0.449	12.4 ± 2.4	11.6 ± 2.7	0.526

All values were shown by mean with standard deviation

HADS scoring system

Tick the bow beside the reply that is closest to how you have been felling in the past week.
Don't take too long over you replies: your immediate is best

D	A		D	A	
		**I feel tense or 'wound up':**			**I feel as if I am slowed down:**
	3	Most of the time	3		Nearly all the time
	2	A lot of the time	2		Very often
	1	From time to time, occasionally	1		Sometimes
	0	Not at all	0		Not at all
		**I still enjoy the things I used to enjoy:**			**I get a sort of frightened feeling like 'butterflies' in the stomach:**
0		Definitely as much		0	Not at all
1		Not quite so much		1	Occasionally
2		Only a little		2	Quite Often
3		Hardly at all		3	Very Often
		**I get a sort of frightened feeling as if something awful is about to happen:**			**I have lost interest in my appearance:**
	3	Very definitely and quite badly	3		Definitely
	2	Yes, but not too badly	2		I don't take as much care as I should
	1	A little, but it doesn't worry me	1		I may not take quite as much care
	0	Not at all	0		I take just as much care as ever
		**I can laugh and see the funny side of things:**			**I feel restless as I have to be on the move:**
0		As much as I always could		3	Very much indeed
1		Not quite so much now		2	Quite a lot
2		Definitely not so much now		1	Not very much

3		Not at all		0	Not at all
		**Worrying thoughts go through my mind:**			**I look forward with enjoyment to things:**
	3	A great deal of the time	0		As much as I ever did
	2	A lot of the time	1		Rather less than I used to
	1	From time to time, but not too often	2		Definitely less than I used to
	0	Only occasionally	3		Hardly at all
		**I feel cheerful:**			**I get sudden feelings of panic:**
3		Not at all		3	Very often indeed
2		Not often		2	Quite often
1		Sometimes		1	Not very often
0		Most of the time		0	Not at all
		**I can sit at ease and feel relaxed:**			**I can enjoy a good book or radio or TV program:**
	0	Definitely	0		Often
	1	Usually	1		Sometimes
	2	Not Often	2		Not often
	3	Not at all	3		Very seldom

Please check you have answered all the questions

Scoring:

Total score: Depression (D) ___________ Anxiety (A) ______________

0-7 = Normal

8-10 = Borderline abnormal (borderline case)

11-21 = Abnormal (case)

Supplementary Table S5. Evaluation of health-related quality of life by using the Sleep Scale from the Medical Outcome Study (MOS-SS)

Outcomes	Placebo (n=15)		*p* value	Red ginseng (n=15)		*p* value
	Week 0	Week 12		Week 0	Week 12	
Sleep disturbance	61.1 ± 15.4	66.9 ± 15.9	0.128	69.1 ± 13.0	70.8 ± 12.4	0.472
Daytime somnolence	74.5 ± 14.8	77.5 ± 16/8	0.342	74.7 ± 17.1	83.0 ± 8.9	0.043
Sleep adequacy	43.8 ± 17.6	40.1 ± 20.9	0.503	44.1 ± 22.6	38.2 ± 24.7	0.345
Snoring	59.4 ± 26.5	63.5 ± 27.3	0.305	64.7 ± 27.6	68.6 ± 28.2	0.624
Awaken short of breath or with headache	90.6 ± 21.9	97.9 ± 5.7	0.102	94.1 ± 11.7	95.1 ± 9.8	0.705
Quality of sleep	6.8 ± 1.7	6.8 ± 1.4	0.660	7.2 ± 1.7	6.9 ± 1.6	0.273
Sleep problems index	64.9 ± 9.3	69.2 ± 6.3	0.098	69.5 ± 5.7	69.2 ± 6.7	0.777

All values were shown by mean with standard deviation

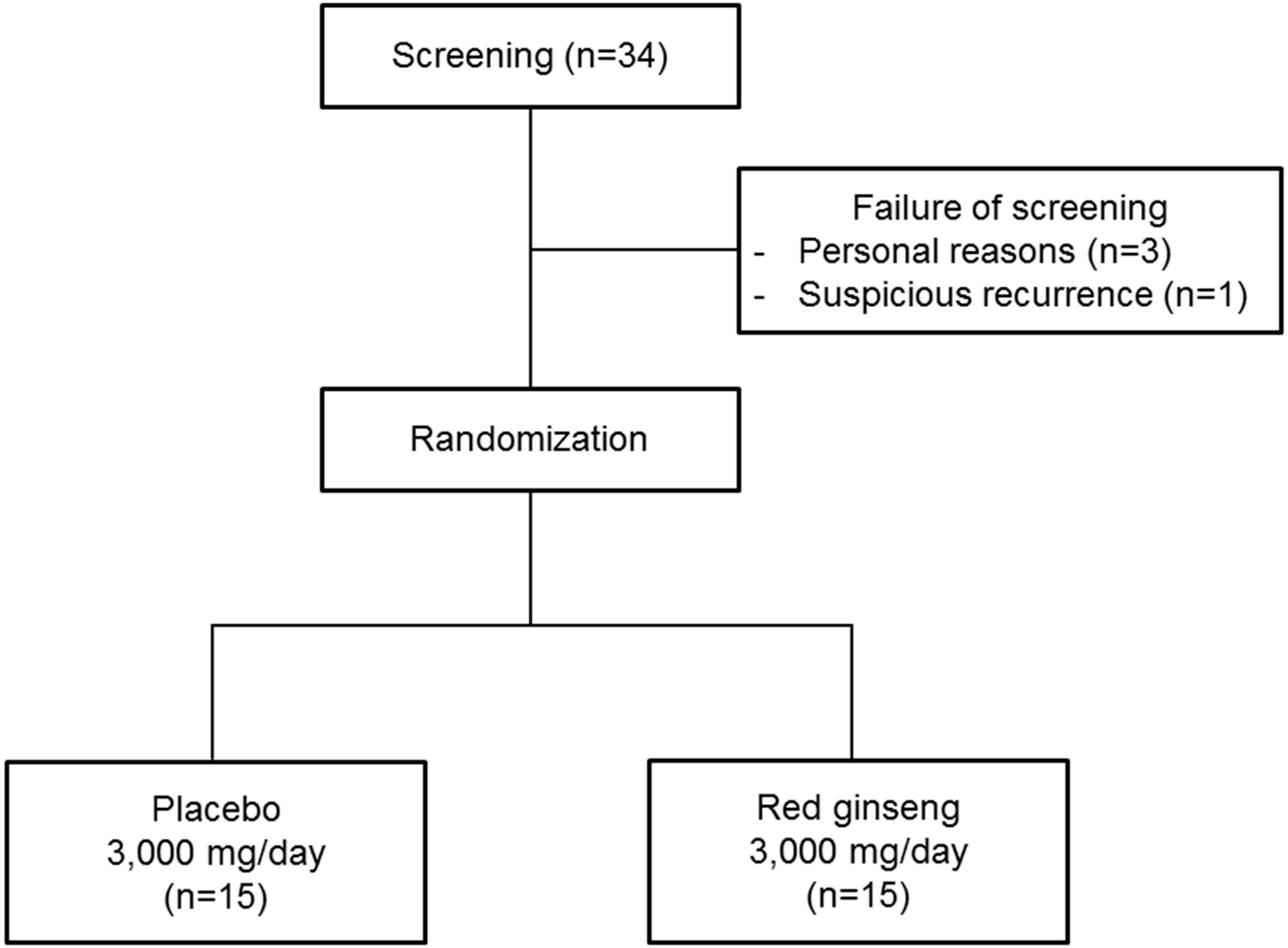

Figure S1. Flow chart of the study design and subject participation.
